# Fabrication of Anti-Reflective Composite Structures on Inverted Pyramids Using Inductively Coupled Plasma Etching

**DOI:** 10.3390/mi16050503

**Published:** 2025-04-26

**Authors:** Zhiwei Fan, Liang Xu, Biyun Zhou, Tao Chen

**Affiliations:** College of Physics and Optoelectronic Engineering, Beijing University of Technology, Beijing 100124, China; zwfang@emails.bjut.edu.cn (Z.F.); xuliang@emails.bjut.edu.cn (L.X.); zbiyun2022@163.com (B.Z.)

**Keywords:** micro-nano composite structures, antireflection, inductively coupled plasma

## Abstract

The anti-reflective properties of silicon surfaces play a pivotal role in determining the light absorption efficiency of various silicon-based optoelectronic devices, with surface micro-nanostructures emerging as a crucial technological approach for achieving enhanced anti-reflection. In this study, inverted pyramid structures were employed as the micron-scale framework, and micro-nano composite structures were successfully prepared using an inductively coupled plasma (ICP) etching system. This paper, mainly focused on the micro-nano fabrication, investigated the effects of gas flow rate ratio (SF_6_:O_2_:C_4_F_8_), ICP power, RF power, and etching time on the surface morphology and reflectance of the composite structures. The results demonstrate that the optimal anti-reflective micro-nano composite structure was achieved under the following conditions: SF_6_ flow rate of 18 sccm, O_2_ flow rate of 9 sccm, C_4_F_8_ flow rate of 4 sccm, ICP power of 300 W, RF power of 5 W, and etching time of 5 min. The average reflectivity of the prepared surface structure was as low as 1.86%.

## 1. Introduction

Silicon, as a core material in the modern semiconductor industry, is widely used in photovoltaic power generation, optoelectronic devices, biosensors, and other fields [1,2,3,4,5,6,7]. However, due to the high refractive index of silicon, intense Fresnel reflection occurs when light transitions from air into silicon, significantly limiting light absorption efficiency. To address this issue, researchers have proposed various strategies to reduce light reflection and enhance light utilization. Currently, the mainstream technical approaches for reducing reflection are anti-reflection coatings and the fabrication of surface micro-nano structures [8]. Anti-reflection coatings are typically designed based on interference effects at specific wavelengths and incident angles, which limits their working angles. Surface micro/nano structure technology has consistently been a focal point of research and interest. Micro-nano structures reduce light reflection and improve absorption efficiency by expanding the contact area, increasing the number of refractions, and changing the equivalent refractive index. In current research, micro-scale structures such as pyramids, V-grooves, and inverted pyramids [9,10] have been proposed. For example, micron-scale pyramid structures can be prepared on silicon surfaces through wet chemical etching or dry etching processes, which effectively reduce light reflection and enhance the conversion efficiency of silicon-based solar cells [11,12]. However, these single micron-scale structures only increase the contact area and the number of refractions between light and the silicon surface, offering limited reduction in reflection. The experimental results demonstrate that, even with optimal treatment, the average reflectance of such textured silicon surfaces remains around 11% across the 300–800 nm wavelength range [13,14].

In 1967, Bernhard [15] first observed periodic structures functioning as antireflective surfaces in nature while studying moths’ eyes. Researchers successfully replicated these moth-eye structures using interference lithography, precisely fabricating regular nanoscale arrays on glass substrates, which pioneered a novel approach to antireflection technology through surface nano-structuring [16,17]. These anti-reflective surfaces must meet a key criterion: the substrate material must be intermixed with air at subwavelength scales [18], such as in porous structures or nanopillar arrays. The fundamental principle of nanostructure-based antireflection can be explained by simple models derived from effective medium theory and graded refractive index theory, originally proposed by J.C.M. Garnett and D.A.G. Bruggeman [19,20]. Based on this, researchers have proposed more complex nanostructures, such as nano-cones, nano-pillar arrays, and biomimetic moth-eye structures [21,22] to form gradient refractive index layers for reducing reflectivity. For instance, in cardiac electrophysiology monitoring, the use of silicon nanoarrays enhances the accuracy of capturing cardiac electrical signals [3]. However, the intricate nature of these structures poses significant challenges for their fabrication. Combining the two approaches, the integration of simple nanostructures onto micron-scale frameworks to form composite structures has emerged as a more effective solution.

In recent years, researchers have proposed and prepared various composite structures, typically using a two-step method: first preparing micron-scale structures and then adding nano-scale structures. Chen et al. [23] utilized laser cleaning-assisted laser ablation technology to prepare multi-scale micro-nano structures in ambient air, demonstrating silicon surfaces with ultralow reflectivity. Yue et al. [24] prepared micro-nano composite structures on silicon surfaces using reactive ion etching (RIE) technology, achieving excellent anti-reflection performance. Yao et al. [25] reported micro-nano composite structures decorated with gold nanoparticles on silicon surfaces, achieving extremely low reflectivity, but with certain limitations in terms of cost and environmental friendliness. In addition to experimental research, extensive theoretical and simulation studies have confirmed that micro- and nanostructures can effectively reduce silicon surface reflectance [22,26,27]. Current research focuses on preparing nano-scale structures on protruding micron-scale structures, resulting in poor mechanical stability of the nano-scale structures. Existing studies have shown that the light-trapping capability of inverted pyramid structures is superior to that of other structures [28], but there are few reports on combining inverted pyramid structures with nano-scale structures to form composite structures.

In this paper, a two-step method was used to prepare composite structures. First, micron-scale inverted pyramid structures were prepared via wet etching. Subsequently, ICP etching was used to create nanostructures on the inverted pyramid surface, integrating micro- and nanostructures to achieve ultra-low reflectance. The effects of gas flow rate ratio (SF_6_:O_2_:C_4_F_8_), ICP power, RF power, and time on the formation of nano-scale structures in inductively coupled plasma (ICP) etching technology were investigated. It should be noted that the chamber size and wafer size can directly affect the etching results. In this study, we used the SI 500 model from SENTECH (Berlin, Germany), a widely used commercial ICP etching system. The chamber volume is 19.2 L, and all wafers used in the experiments measured 2 × 2 cm^2^. The surface morphology was characterized using scanning electron microscopy (SEM), and the anti-reflection performance of the composite structures was evaluated using a spectrophotometer.

## 2. Materials and Methods

### 2.1. Materials

The materials used were monocrystalline silicon wafers (P-type, (100) orientation, resistivity 1–10 Ω·cm, 20 mm × 20 mm, Suzhou Yan Cai Micro-Nano Technology Co., Ltd., Suzhou, China); Photoresist (SPR-955, Futurrex, Saginaw, TX, USA); potassium hydroxide (KOH); isopropyl alcohol (IPA); hydrogen peroxide (H_2_O_2_); hydrochloric acid (HCl); ammonium hydroxide (NH_4_OH); hydrofluoric acid (HF); deionized water; SF_6_ (99.999%); O_2_ (99.999%); C_4_F_8_ (99.999%) CHF_3_ (99.999%); CF_4_ (99.999%); and Ar (99.999%).

### 2.2. Preparation of Composite Structures

The inverted pyramid structures were prepared using photolithographic patterning-assisted wet etching. First, the standard RCA cleaning method was applied to clean the silicon wafer. Organic contaminants and particulate matter were removed by immersion in a NH_4_OH:H_2_O_2_:H_2_O (1:1:5) solution at 75 °C; Metallic impurities were eliminated in a HCl:H_2_O_2_:H_2_O (1:1:6) solution at 75 °C. Finally, the wafer was immersed in a HF:H_2_O (1:50) solution at room temperature to remove the native oxide layer. After each step, deionized water (DI water) was used for thorough rinsing to prevent cross-contamination. After cleaning, a 150-nm-thick SiO_2_ layer was thermally grown on the wafer surface. Spin-coating SPR-955 Photoresist and patterning periodic square arrays (10-μm features with 2-μm spacing) was performed via photolithography. The SiO_2_ layer within the square patterns was removed by dry etching (etching gases: 20 sccm CHF_3_, 40 sccm CF_4_, 10 sccm Ar; etching time: 35 s). The patterned silicon wafers were immersed in a 30% KOH solution doped with 10% IPA and etched in a water bath at 80 °C. A magnetic stirrer was used to maintain uniform concentration and temperature of the solution. The measured etching rate was 0.941 µm/min. The IPA additive played a crucial role in reducing surface tension, optimizing etchant distribution, and enhancing hydrogen gas release [29]. After etching for 8 min and 30 s, the wafers were removed, rinsed with deionized water, and immersed in a 5% HF solution for 30 s to remove the SiO_2_ mask and residual oxides. The wafers were rinsed again with deionized water, dried with nitrogen, and stored in a nitrogen cabinet.

Nanostructures were prepared using inductively coupled plasma (ICP) etching technology. The silicon wafers with prepared inverted pyramid structures were placed in the sample loading chamber of the ICP etching machine. The chamber was evacuated to match the pressure of the main chamber, and the samples were then transferred for processing. The gas flow rate ratio (SF_6_:O_2_:C_4_F_8_), ICP power, RF power, and etching time were adjusted to study the effects of these parameters on the surface morphology and anti-reflection performance of the samples.

The surface morphology of the composite structures was observed using a field emission scanning electron microscope (Verios 460, FEI, Hillsboro, OR, USA). The total surface reflectance of different samples was measured using a spectrophotometer (UV3600, Shimadzu, Kyoto, Japan, equipped with an integrating sphere) in the wavelength range of 300–800 nm.

## 3. Results and Discussion

The ICP system primarily relies on high-frequency power to excite inert gas, generating high-density plasma that decomposes reactive gases (SF_6_:O_2_:C_4_F_8_) into active radicals and ions. SF_6_ decomposition provides fluorine radicals (F) to etch silicon, O_2_ decomposes into oxygen atoms (O) that react with silicon (Si) to form a passivation layer, and C_4_F_8_ decomposes to generate fluorocarbon polymers (CF_x_), forming a sidewall protection layer to enhance anisotropy [30]. The chemical reactions are as follows:

(1) $${SF}_{6}+e^{-}\to {SF}_{5}^{+}+F+{2e}^{-}$$

(2) $$Si+{4F}^{-}\to {SiF}_{4}↑$$

(3) $$Si+2O\to {SiO}_{2}$$

The gaseous byproducts generated during the etching reaction are evacuated by the vacuum pump of the ICP system, ensuring the continuous progress of the reaction.

To investigate the effects of different process parameters on the etching results and optical performance, a series of experiments was systematically designed by varying the gas flow rate, C_4_F_8_ concentration, ICP power, RF power, and etching duration. The detailed parameters for each sample, along with their corresponding average reflectance values, are presented in Table 1. Sample 0 corresponds to the inverted pyramid structure prepared by wet etching, while the remaining samples represent the composite structures.

**Figure 1:**
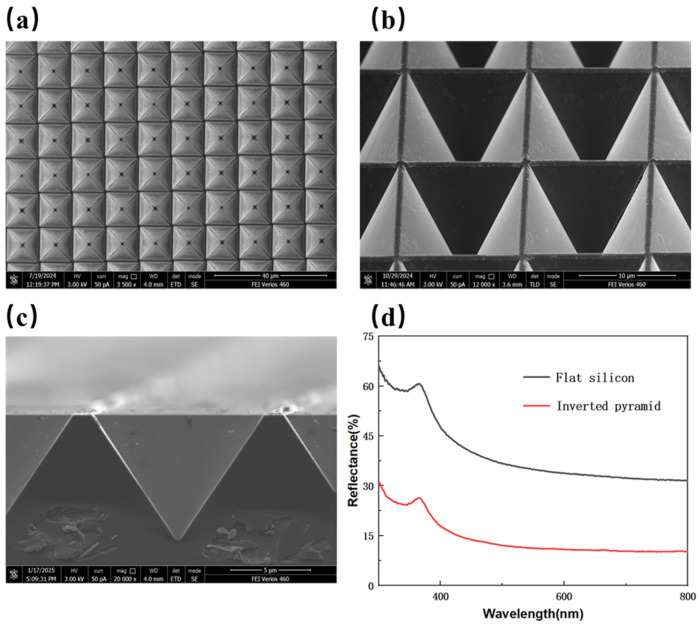
(**a**) SEM image of the inverted pyramid structure; (**b**) 45° tilted view; (**c**) Cross-sectional view; (**d**) Reflectivity spectra of planar silicon and inverted pyramid-structured silicon.

Figure 1a is the SEM image of the inverted pyramid structure prepared by wet etching, indicating that the inverted pyramid structures on the silicon surface are uniformly arranged, structurally intact, and highly reproducible. Figure 1b,c show the 45° tilted and cross-sectional SEM images of the inverted pyramid structure, respectively, revealing a smooth internal surface without residues, confirming the successful preparation of the structure. Figure 1d displays the reflectivity of planar silicon and inverted pyramid-structured silicon, showing that the reflectance of the inverted pyramid-structured silicon is significantly lower than that of planar silicon, with a notable improvement in anti-reflection performance. The average reflectance decreased from 39.80% to 13.85%, a reduction of 68.72%. Compared with previously reported findings [13,14], the average reflectance is higher, which is attributed to the presence of gaps between the inverted pyramid structures prepared by this method, resulting in a reduced contact area. It can also be observed that a reflectance peak appears around 380 nm. This phenomenon occurs because one of the band gaps of p-type silicon is 3.35 eV, where the photon energy is close to the transition energy, causing a peak in the refractive index [31]. When light is normally incident from air onto the silicon surface, the reflectance coefficient (*R*) can be expressed as:

(4) $$R=\frac{{\left(n-1\right)}^{2}+k^{2}}{{\left(n+1\right)}^{2}+k^{2}}$$

where *n* is the refractive index, and *k* is the extinction coefficient. Thus, when the refractive index exhibits a peak, the reflectance coefficient also increases, leading to the observed peak around 380 nm. The inverted pyramid-structured substrate demonstrates excellent anti-reflection properties, providing a foundation for subsequent nanostructure preparation and further reduction of reflectance.

**Figure 2:**
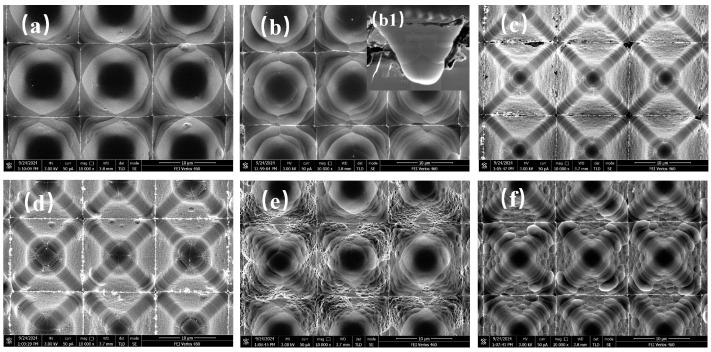
SEM images of surfaces etched with different SF_6_:O_2_ rate ratios. SF_6_:O_2_ (sccm): (**a**) 18:3; (**b**) 18:6; (**c**) 18:9; (**d**) 18:12; (**e**) 18:15; (**f**) 18:18; (**b1**) Cross-sectional image.

The flow rate ratio of SF_6_ to O_2_ is a critical factor in the etching process. By controlling the etching time to 5 min, ICP power to 300 W, and RF power to 0 W, the formation of nanostructures under different flow rate ratios was investigated. Figure 2 is the SEM images of surfaces etched with different SF_6_:O_2_ flow rate ratios. The black cross-shaped lines in the images result from localized charge accumulation under electron beam irradiation. This accumulation is more pronounced at the structural junctions, leading to the appearance of black cross-shaped lines in the images. From Figure 2a,b, it can be observed that at lower O_2_ ratios, the inverted pyramid structures are damaged, and no significant nanostructures are formed. The sidewall profiles tend to become rounded. This is because the passivation effect of O_2_ at low ratios cannot compete with the etching effect of SF_6_. Higher SF_6_ concentrations result in faster etching rates, but insufficient O_2_ content leads to inadequate formation of an oxide mask, resulting in poor anisotropy. From Figure 2(b1), it can be observed that arc-shaped stepped structures are formed inside the structure, while petal-like patterns appear on the surface. This phenomenon is attributed to the uneven distribution of the mixed SF_6_ and O_2_ gases within the inverted pyramid structure during the etching process, leading to locally enhanced or weakened etching. Specifically, within the inverted pyramid structure, the plasma density decreases towards the bottom. Without applied RF power, this results in different etching rates between the upper and lower parts of the structure, ultimately leading to the formation of petal-like morphology [32,33].

As the O_2_ content increases, the etching results are shown in Figure 2c,d. The inverted pyramid profiles remain intact, and the sidewall angles are preserved, but no significant nanostructures are formed, with only small protrusions on the sidewall surfaces. Further increasing the O_2_ ratio, as shown in Figure 2e,f, shows deposition at grain boundaries and the bottom due to the accelerated formation of the oxide mask caused by the higher O_2_ concentration, which inhibits the etching rate. Throughout the etching process, fluorine ions from SF_6_ provide chemical etching capability, while O_2_ generates an oxide mask protective layer [34]. However, at room temperature, no effective nanostructures are formed under all condition cases. C_4_F_8_ was introduced in subsequent experiments to balance passivation and etching effects. C_4_F_8_ generates fluorocarbon films that deposit on the sidewalls of nanostructures, effectively suppressing lateral etching and protecting the nanostructures from damage.

**Figure 3:**
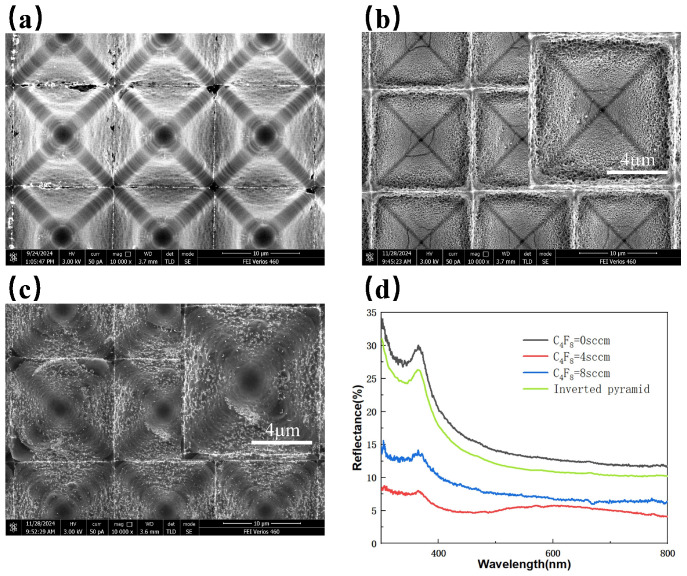
SEM images of composite structures under different C_4_F_8_ flow rates: (**a**) 0 sccm; (**b**) 4 sccm; (**c**) 8 sccm; (**d**) Reflectivity spectra of composite-structured silicon wafers under different C_4_F_8_ flow rates.

**Figure 4:**
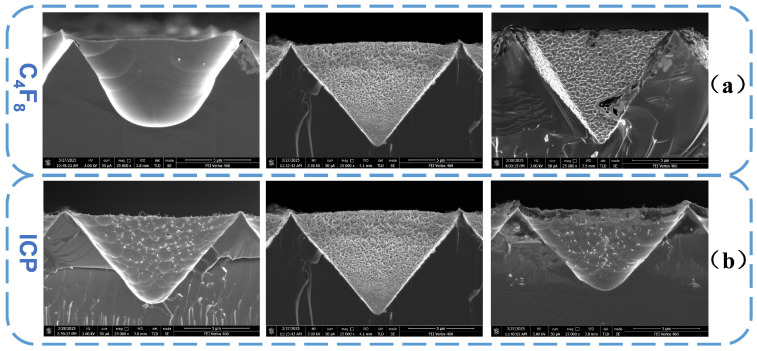
Cross-sectional images of structures under different etching conditions: (**a**) C_4_F_8_ (sccm) from left to right: 0, 4, 8; (**b**) ICP power (W) from left to right: 150, 300, 450.

With SF_6_ fixed at 18 sccm, O_2_ at 9 sccm, ICP power at 300 W, RF power at 0 W, and etching time at 5 min, Figure 3a–c show the SEM images of composite structures surfaces under C_4_F_8_ flow rates of 0 sccm, 4 sccm, and 8 sccm, respectively, while Figure 4a corresponds to cross-sectional images of the composite structures at different C_4_F_8_ flow rates. Figure 3a depicts the etching morphology without the introduction of C_4_F_8_, where no effective nanostructures are formed. The cross-sectional image reveals that the bottom of the inverted pyramid structure is damaged and presents a curved profile. Figure 3b shows the etching morphology with the addition of 4 sccm C_4_F_8_, where hollow-like nanostructures are formed. The cross-sectional view indicates that the inverted pyramid structure remains relatively intact, with nanostructures forming throughout the interior. Figure 3c presents the etching morphology with 8 sccm C_4_F_8_, where the nanostructures on the sidewalls of the inverted pyramid structure are not fully formed. The cross-sectional image reveals the presence of ridge-like protrusions, while the nanostructures at the bottom disappear and those on the sidewalls remain incomplete. This is because the introduction of C_4_F_8_ generates fluorocarbon films that deposit on the sidewalls of the nanostructures, enhancing anisotropic etching. However, excessive C_4_F_8_ leads to the over-formation of fluorocarbon compounds, resulting in residues that inhibit etching. Figure 3d compares the reflectivity spectra of composite-structured silicon wafers under different C_4_F_8_ flow rates. The average reflectance values are 16.3%, 5.56%, and 8.71% for C_4_F_8_ flow rates of 0 sccm, 4 sccm, and 8 sccm, respectively. Without the addition of C_4_F_8_, the average reflectance increases by 2.45% due to the lack of effective nanostructures, which causes the inverted pyramid structures to be etched and their depth reduced, thereby diminishing their anti-reflection capability.

**Figure 5:**
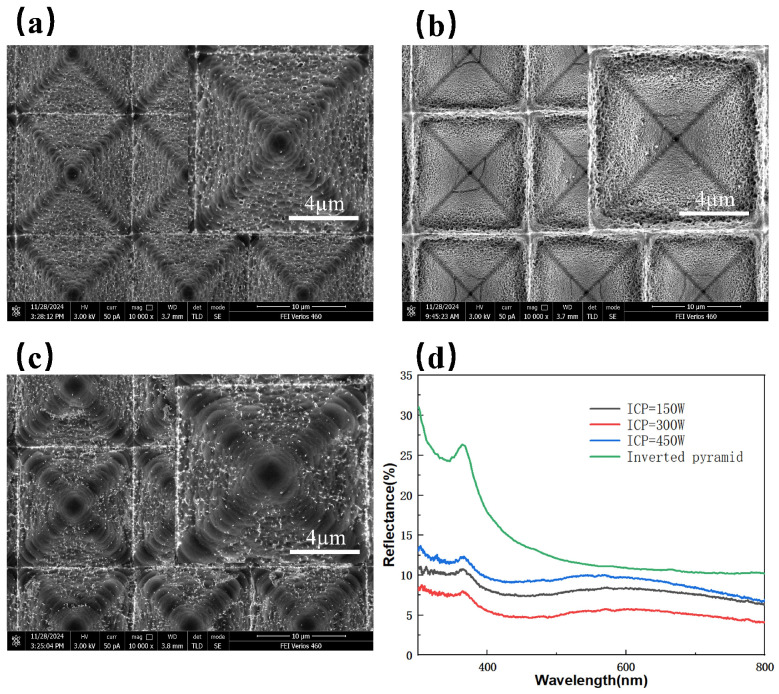
SEM images of composite structures under different ICP powers: (**a**) 150 W; (**b**) 300 W; (**c**) 450 W; (**d**) Reflectivity spectra of composite-structured silicon wafers under different ICP powers.

ICP power directly affects the plasma density. With SF_6_ fixed at 18 sccm, O_2_ at 9 sccm, C_4_F_8_ at 4 sccm, RF power at 0 W, and etching time at 5 min. Figure 5a–c show SEM images of the composite structure surfaces under different ICP power levels, while Figure 4b corresponds to their cross-sectional views. Figure 5a shows the SEM image at an ICP power of 150 W, where small hill-like protrusions are formed on the sidewalls of the inverted pyramid structures, but the nanostructures are sparse due to low plasma density and insufficient etching reactions. When the power is increased to 300 W, as shown in Figure 5b, a dynamic balance between etching and passivation ion densities in the plasma is achieved, resulting in the formation of hollow-like nanostructures on the surface, which effectively reduce reflectance. Further increasing the power to 450 W, as shown in Figure 5c, reveals polymer deposition at the grain boundaries of the structures due to the excessive heat generated at high power [35], which damages the nanostructures. The cross-sectional images in Figure 4b reveal that, as the ICP power increased, the depth of the inverted pyramid structures gradually decreased. High ICP power accelerated the etching rate. Figure 5d compares the reflectivity spectra of composite structures under different ICP powers. The average reflectance values are 8.71%, 5.56%, and 9.89% for ICP powers of 150 W, 300 W, and 450 W, respectively. This is because the combination of nanostructures and microstructures creates a gradual refractive index transition from air to the silicon substrate, effectively reducing reflectance. This effect is particularly pronounced in the 300 to 550 nm wavelength range, as shorter wavelengths are more sensitive to changes in refractive index.

**Figure 6:**
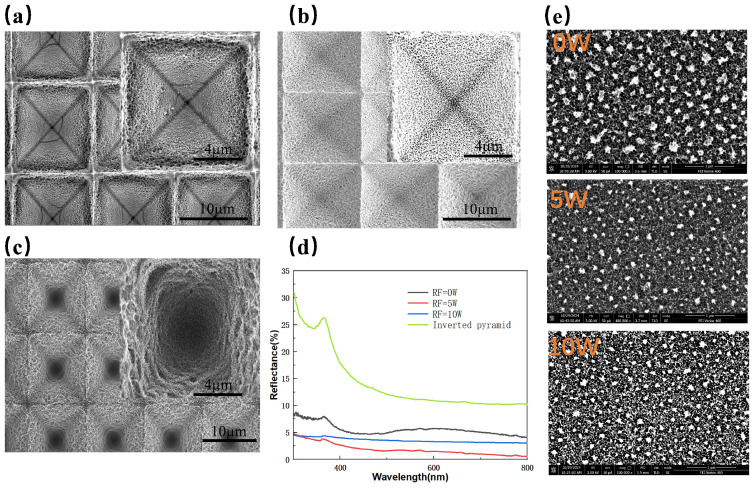
SEM images of composite structures under different RF powers: (**a**) 0 W; (**b**) 5 W; (**c**) 10 W; (**d**) Reflectivity spectra of composite-structured silicon wafers under different RF powers; (**e**) SEM images of etched flat silicon wafers under different RF power conditions.

The radio frequency (RF) power primarily affects the energy of ions and the directionality of etching during the formation of nanostructures. With SF_6_ fixed at 18 sccm, O_2_ at 9 sccm, C_4_F_8_ at 4 sccm, ICP power at 300 W, and etching time at 5 min. Figure 6b shows the SEM image of the composite structure surface morphology at an RF power of 5 W. Compared to the sample without RF power, circular pore-like nanostructures are formed, and the overall morphology of the composite structure is more complete, with more regular nanostructures. This is because the application of RF power provides directional energy to the radical ions, resulting in better anisotropy. Figure 6c shows the SEM image of the composite structure surface morphology at an RF power of 10 W. As the RF power further increases, the radical ions acquire more energy, resulting in the degradation of nanostructures. However, the depth of the inverted pyramid structure increases, which also contributes to anti-reflection. Figure 6d compares the reflectivity spectra of composite-structured silicon wafers at RF powers of 0 W, 5 W, and 10 W, with average reflectance values of 5.56%, 1.86%, and 3.55%, respectively. When the RF power is 5 W, the reflectance is only 1.86%, compared to 8.31% reported by Liu et al. for samples prepared via metal-assisted chemical etching [36], demonstrating excellent antireflective performance. According to Fresnel’s theory, when the refractive indices of two media are more similar, the reflection is reduced. Therefore, a gradual refractive index distribution from air to substrate is formed on the material’s surface, preventing abrupt changes in refractive index and minimizing light reflection at the interface. For nanostructures, this can be likened to a multi-layer film with gradually decreasing refractive indices, where the refractive indices between the two equivalent media are very close, resulting in an extremely low surface reflectance [26]. We also conducted dry etching experiments on planar silicon wafers to compare the performance of different surface structures. As shown in Figure 6e, the etching results under various RF powers on planar silicon reveal significant fluorocarbon polymer deposition on the surface, which is in stark contrast to the results observed on inverted pyramid structures. This discrepancy may be attributed to the fact that etching reactions on planar surfaces tend to promote surface accumulation. In contrast, the sidewalls of the inverted pyramids are (111) crystal planes with an inclination angle, making it more difficult for reaction byproducts to adhere and accumulate. Additionally, ions incident vertically can bombard the sidewalls, further reducing deposition. It is also possible that localized gas flow disturbances within the inverted pyramid structures enhance the exchange of reactive gases and byproducts, thereby suppressing polymer buildup.

**Figure 7:**
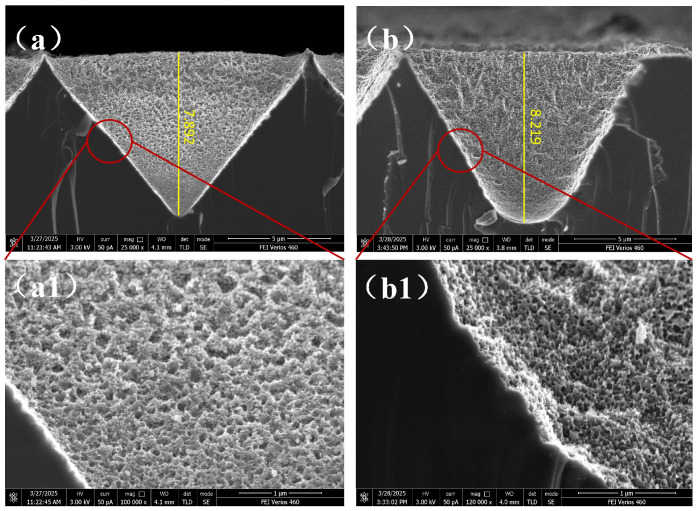
Cross-sectional images of composite structures at different RF power levels: (**a**) 0 W; (**b**) 10 W; (**a1**,**b1**) Locally magnified images.

Figure 7 presents the cross-sectional SEM images of the structures shown in Figure 6a,c. It clearly reveals the formation of a hollow multi-layered porous structure on the inverted pyramid structures. Upon applying RF power, the depth of the inverted pyramid structures increases from 7.892 µm to 8.219 µm, and the bottom transforms into an arc shape. This indicates that plasma, due to its initial velocity, can more easily reach the bottom of the structures. The confined space at the bottom restricts plasma flow, accelerating the etching rate, while physical bombardment further deepens the etched structures. However, the narrow bottom space also hinders the diffusion of etching byproducts, leading to enhanced lateral etching, which transforms the bottom morphology from sharp to arc-shaped. As seen in Figure 7(a1,b1), the hollow porous structures formed under RF power exhibit greater depth and smaller pore sizes, suggesting that RF power promotes vertical etching and enhances etching anisotropy.

**Figure 8:**
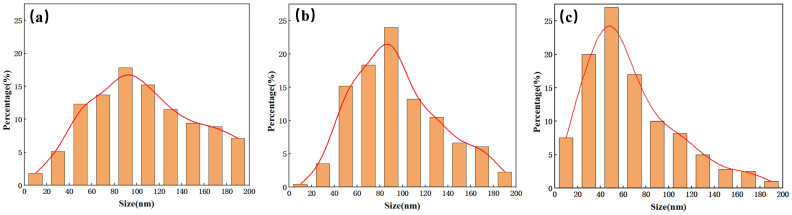
Diameter distribution of nanostructures under different RF power levels: (**a**) 0 W; (**b**) 5 W; (**c**) 10 W.

**Figure 9:**
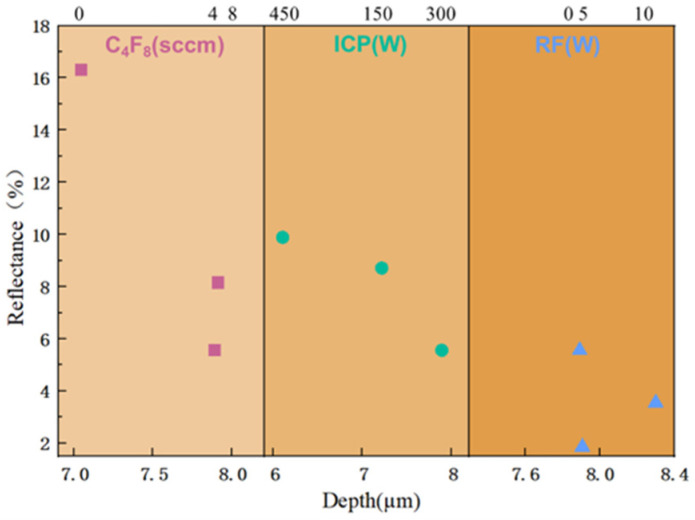
Scatter plot of the depth of inverted pyramid structures and average reflectance under different etching parameters.

Figure 8 shows the pore size distribution of nanostructures under different RF power levels. As RF power increases, the characteristic size of the nanostructures decreases, but it remains primarily within the 20–200 nm range. The characteristic size of 20–200 nm is significantly smaller than the wavelength of visible light, forming a uniform effective medium layer. Additionally, subwavelength structures facilitate light coupling into silicon through scattering and interference effects [18]. As observed in Figure 6d, the reflectance of all three composite structures decreases significantly within the 300–800 nm wavelength range, with a particularly large reduction in the reflection peak around 380 nm when RF power is 10 W. This phenomenon occurs because, at a wavelength of approximately 380 nm, silicon has a refractive index of 6.709. According to the relation λ/n_Si_, when the characteristic size of the nanostructures is around 54 nm, Mie resonance is excited, leading to a reduction in reflectance within this wavelength range. Figure 8c shows that, at an RF power of 10 W, most of the nanostructures have a characteristic size around 50 nm, which explains the significant decrease in reflectance near 380 nm [37]. Figure 9 presents a scatter plot of the depth of inverted pyramid structures and their average reflectance under different etching parameters. It reveals that, when nanostructure formation is suboptimal, deeper inverted pyramid structures make it more difficult for light to escape from the structure’s bottom, resulting in a lower average reflectance.

**Figure 10:**
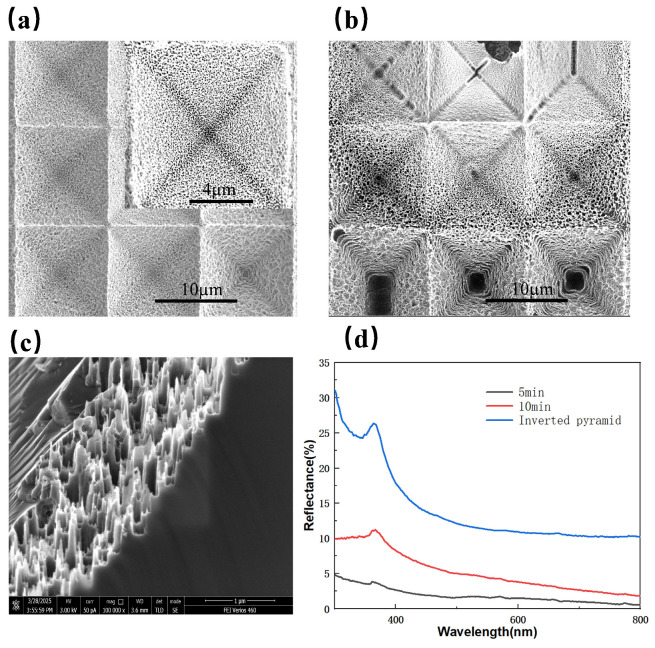
SEM images of composite structures under different etching times: (**a**) 5 min; (**b**) 10 min; (**c**) Cross-sectional image at 5 min; (**d**) Reflectivity spectra of composite-structured silicon wafers at different times.

Figure 10a,b show the surface morphology of composite structures under different etching times. It is evident that, as the etching time extends to 10 min, the inverted pyramid structures undergo damage, and the pores within the nanostructures progressively expand, with some being compromised by lateral etching, ultimately resulting in diminished anti-reflection performance. Figure 10c shows the cross-sectional image of the structures after 5 min of etching, revealing the formation of porous and nano-cone-like structures with a high aspect ratio, which can induce a gradient refractive index effect. From the reflection spectrum in Figure 10d, it can be observed that, when the etching time reaches 10 min, the reflectance increases significantly, with an average value of 5.24%.

## 4. Conclusions

Using ICP etching technology, nanostructures were prepared on silicon wafers with periodic inverted pyramid structures of 10 μm in size and 2 μm in spacing to form composite structures. Research indicates that, at room temperature, effective nanostructures cannot be generated using only SF_6_ and O_2_ as etching gases, and an appropriate amount of C_4_F_8_ is required to balance the reaction. The optimal anti-reflection composite structure was achieved under the conditions of SF_6_ flow rate at 18 sccm, O_2_ at 9 sccm, C_4_F_8_ at 4 sccm, ICP power at 300 W, RF power at 5 W, and etching time of 5 min. This structure exhibited an extremely low reflectance of 1.86% in the wavelength range of 300–800 nm, representing a reduction in average reflectance by 37.94% compared to planar silicon wafers.

## Figures and Tables

**Table 1:** Sample parameters under different experimental conditions.

Sample	SF_6_:O_2_ (sccm)	C_4_F_8_ (sccm)	ICP (W)	RF (W)	Time (min)	Average Reflectance 300–800 nm (%)
0	0	0	0	0	0	13.85
1	18:3	0	300	0	5	/
2	18:6	0	300	0	5	/
3	18:9	0	300	0	5	16.3
4	18:12	0	300	0	5	/
5	18:15	0	300	0	5	/
6	18:18	0	300	0	5	/
7	18:9	4	300	0	5	5.56
8	18:9	8	300	0	5	8.15
9	18:9	4	150	0	5	8.71
10	18:9	4	450	0	5	9.89
11	18:9	4	300	5	5	1.86
12	18:9	4	300	10	5	3.55
13	18:9	4	300	5	10	5.24

“/” indicates that the measurement was not performed, as the sample was used for the formation of effective nanostructures.

## Data Availability

The original contributions presented in the study are included in the article, further inquiries can be directed to the corresponding author.

## References

[1] Pera D.M., Costa I., Serra F., Gaspar G., Lobato K., Serra J.M., Silva J.A. (2023). Development of a metal-assisted chemical etching method to improve light-capture in monocrystalline silicon solar cells. Sol. Energy Mater. Sol. Cells.

[2] Serpenguzel A., Inanc¸ I., Carey J., Mazur E., Kurt A. (2008). Luminescence of black silicon. J. Nanophotonics.

[3] Fang H., Yu K.J., Gloschat C., Yang Z., Song E., Chiang C.-H., Zhao J., Won S.M., Xu S., Trumpis M. (2017). Capacitively coupled arrays of multiplexed flexible silicon transistors for long-term cardiac electrophysiology. Nat. Biomed. Eng..

[4] Ban J., Lu Y., Lu J., Jia K., Luo M., Zhou Y., Wang D., Piao L. (2024). Highly sensitive stretchable fiber-based temperature sensor enhanced by surface-chemically modified silver nanowires. Chem. Eng. J..

[5] Tong L., Wan J., Xiao K., Liu J., Ma J., Guo X., Zhou L., Chen X., Xia Y., Dai S. (2023). Heterogeneous complementary field-effect transistors based on silicon and molybdenum disul-fide. Nat. Electron..

[6] Bisogni M.G., Del Guerra A., Belcari N. (2019). Medical applications of silicon photomultipliers. Nucl. Instrum. Methods Phys. Res. Sect. A Accel. Spectrometers Detect. Assoc. Equip..

[7] Chen L., Zhang S., Duan Y., Song X., Chang M., Feng W., Chen Y. (2024). Silicon-containing nanomedicine and biomaterials: Materials chemistry, multi-dimensional design, and biomedical application. Chem. Soc. Rev..

[8] Yeo R.J., Wu W.Y., Tomczak N., Ji R., Wang S., Wang X., Kong J., Liu H., Goh K.E.J., Xu J. (2023). Tailoring surface reflectance through nanostructured materials design for energy-efficient applications. Mater. Today Chem..

[9] Almenabawy S., Zhang Y., Flood A., Prinja R., Kherani N.P. (2022). Nanometer-mesa inverted-pyramid photonic crystals for thin silicon solar cells. ACS Appl. Energy Mater..

[10] Feng B., Chen W., Xing G., Chen X., Li H., Sun Z., Zhang Y., Liu Y., Du X. (2024). Influence of inverted pyramid texturization on front metallization and performance of crystalline silicon solar cells. Sol. Energy Mater. Sol. Cells.

[11] Yu R., Lin Q., Leung S.-F., Fan Z. (2012). Nanomaterials and nanostructures for efficient light absorption and photovoltaics. Nano Energy.

[12] Liu H., Du Y., Yin X., Bai M., Liu W. (2022). Micro/nanostructures for light trapping in monocrystalline silicon solar cells. J. Na-Nomaterials.

[13] Wang Y., Yang L., Liu Y., Mei Z., Chen W., Li J., Liang H., Kuznetsov A., Xiaolong D. (2015). Maskless inverted pyramid texturization of silicon. Sci. Rep..

[14] Abdullah M.F., Hashim A.M. (2019). Reflectance Characteristics of Silicon Surface Fabricated with the Arrays of Uniform Inverted Pyramid Microstructures in UV-Visible Range. Sains Malays..

[15] Bernhard C.G. (1967). Structural and functional adaptation in a visual system. Endeavour.

[16] Peng K., Wu Y., Fang H., Zhong X., Xu Y., Zhu J. (2005). Uniform, axial-orientation alignment of one-dimensional single-crystal silicon nanostructure arrays. Angew. Chem.Int. Ed..

[17] Nichkalo S., Druzhinin A., Evtukh A., Bratus’ O., Steblova O. (2017). Silicon Nanostructures Produced by Modified MacEtch Method for Antireflective Si Surface. Nanoscale Res. Lett..

[18] Gombert A., Glaubitt W., Rose K., Dreibholz J., Bläsi B., Heinzel A., Sporn D., Döll W., Wittwer V. (1999). Subwavelength-structured antireflective surfaces on glass. Thin Solid Films.

[19] Maxwell G.J., Garnett B.A. (1904). Colours in metal glasses and in metallic films. Philos. Trans. R. Soc. Lond. Ser. A Contain. Pap. Math. Phys. Character.

[20] Bruggeman V.D.A.G. (1935). Berechnung verschiedener physikalischer Konstanten von heterogenen Substanzen. I. Dielektri-zitätskonstanten und Leitfähigkeiten der Mischkörper aus isotropen Substanzen. Ann. Der Phys..

[21] Rahman T., Navarro-Cía M., Fobelets K. (2014). High density micro-pyramids with silicon nanowire array for photovoltaic applica-tions. Nanotechnology.

[22] Lan J., Yang Y., Hu S. (2021). Numerical Study on Broadband Antireflection of Moth-Eye Nanostructured Polymer Film with Flexible Polyethylene Terephthalate Substrate. Nanomaterials.

[23] Chen T., Wang W., Tao T., Pan A., Mei X. (2020). Multi-scale micro-nano structures prepared by laser cleaning assisted laser ablation for broadband ultralow reflectivity silicon surfaces in ambient air. Appl. Surf. Sci..

[24] Yue Z., Shen H., Jiang Y. (2013). Antireflective nanostructures fabricated by reactive ion etching method on pyramid-structured silicon surface. Appl. Surf. Sci..

[25] Yao C., Liu Y., Niu J., Lu C., Li H., Xie C. (2024). Micro/nano-hybrid hierarchical structure of black silicon decorated with gold nanoparticles for ultralow broadband reflectivity (<1%). Appl. Surf. Sci..

[26] Chattopadhyay S., Huang Y., Jen Y., Ganguly A., Chen K., Chen L. (2010). Anti-reflecting and photonic nanostructures. Mater. Sci. Eng. R Rep..

[27] Park H., Shin D., Kang G., Baek S., Kim K., Padilla W.J. (2011). Broadband optical antireflection enhancement by integrating antireflective nanoislands with silicon nanoconical-frustum arrays. Adv. Mater..

[28] Sun Z., Chen W., Zhang X., Xu M., Xing G., Chen X., Feng B., Li H., Ma J., Wang Y. (2023). Chain pyramid texturization for better light trapping and efficiency of silicon solar cells. Sol. Energy Mater. Sol. Cells.

[29] Burham N., Hamzah A., Majlis B.Y. Effect of isopropyl alcohol (IPA) on etching rate and surface roughness of silicon etched in KOH solution. Proceedings of the 2015 IEEE Regional Symposium on Micro and Nanoelectronics (RSM).

[30] Susarrey-Arce A., Marín Á.G., Nair H., Lefferts L., Gardeniers J.G.E., Lohse D., van Houselt A. (2012). Absence of an evaporation-driven wetting transition on omniphobic surfaces. Soft Matter.

[31] El-Zaiat E.S.Y., Youssef G.M. (2015). Dispersive parameters for complex refractive index of p-and n-type silicon from spectrophotometric measurements in spectral range 200–2500 nm. Opt. Laser Technol..

[32] Legtenberg R., Jansen H., de Boer M., Elwenspoek M. (1995). Anisotropic reactive ion etching of silicon using SF_6_/O_2_/CHF_3_ gas mixtures. J. Electrochem. Soc..

[33] Bates R.L., Stephan Thamban P.L., Goeckner M.J., Overzet L. (2014). Silicon Etch Using SF_6_/C_4_F_8_/Ar Gas Mixtures. J. Vac. Sci. Technol. A.

[34] Sainiemi L., Jokinen V., Shah A., Shpak M., Aura S., Suvanto P., Franssila S. (2011). Non-reflecting silicon and polymer surfaces by plasma etching and replication. Adv. Mater..

[35] Tinck S., Tillocher T., Georgieva V., Dussart R., Neyts E., Bogaerts A. (2017). Concurrent effects of wafer temperature and oxygen fraction on cryogenic silicon etching with SF_6_/O_2_ plasmas. Plasma Process. Polym..

[36] Liu Y., Dong K., Bian L., Guan Z. (2021). One-step fabrication of inverted pyramid textured silicon wafers via silver-assisted chemical etching combing with synergism of polyvinylpyrrolidone (PVP). Crystals.

[37] Bezares F.J., Long J.P., Glembocki O.J., Guo J., Rendell R.W., Kasica R., Shirey L., Owrutsky J.C., Caldwell J.D. (2013). Mie resonance-enhanced light absorption in periodic silicon nanopillar arrays. Opt. Express.

